# Risk Analysis of Earth-Rock Dam Failures Based on Fuzzy Event Tree Method

**DOI:** 10.3390/ijerph15050886

**Published:** 2018-04-29

**Authors:** Xiao Fu, Chong-Shi Gu, Huai-Zhi Su, Xiang-Nan Qin

**Affiliations:** 1 State Key Laboratory of Hydrology-Water Resources and Hydraulic Engineering, Hohai University, Nanjing 210098, China; fuxiaohhu@163.com (X.F.); su_huaizhi@hhu.edu.cn (H.-Z.S.); Qin_xn@163.com (X.-N.Q.); 2 National Engineering Research Center of Water Resources Efficient Utilization and Engineering Safety, Hohai University, Nanjing 210098, China; 3 College of Water Conservancy and Hydropower Engineering, Hohai University, Nanjing 210098, China

**Keywords:** earth-rock dam, risk analysis, dam failure probability, life loss, ETA method, fuzzy set theory

## Abstract

Earth-rock dams make up a large proportion of the dams in China, and their failures can induce great risks. In this paper, the risks associated with earth-rock dam failure are analyzed from two aspects: the probability of a dam failure and the resulting life loss. An event tree analysis method based on fuzzy set theory is proposed to calculate the dam failure probability. The life loss associated with dam failure is summarized and refined to be suitable for Chinese dams from previous studies. The proposed method and model are applied to one reservoir dam in Jiangxi province. Both engineering and non-engineering measures are proposed to reduce the risk. The risk analysis of the dam failure has essential significance for reducing dam failure probability and improving dam risk management level.

## 1. Introduction

Earth-rock dams account for more than 90% of all of the 90,000 reservoirs in China, among which nearly 30,000 reservoirs are in operation with defects. The primary problem for decision-makers to solve is how to protect the safety of reservoirs and use the nation’s most limited funds to reinforce the most in-need reservoirs [1]. The use of risk analysis to manage these dams has become an urgent problem in the dam industry [2,3,4]. Dam failure is a low-probability social catastrophic factor, which is extremely harmful. There are great numbers of downstream residents of reservoirs in China. The life loss will be great and intolerable once a dam is damaged. According to statistics, the annual average dam failure rate in China is 8.761 × 10^−4^. In the 20th century, the average annual dam failure rate of the world was 2.0 × 10^−4^, regardless of war-related reasons, which means the probability of dam failure in China is relatively high [5].

A number of studies are dedicated to investigating dam failures. Hartford et al. [6] provided a contemporary description of evolving techniques for risk-based dam safety management. They presented some new approaches (e.g., ETA method and comprehensive sections on consequence analysis), which are necessary for the estimation of risk and the planning of emergency preparedness. Rong-Yong and Zong-Kun et al. [7,8] analyzed the overflow fuzzy risk on earth dams, which makes the calculation of dam failure probability more reasonable. Graham [9] conducted an extensive evaluation of dam failures and the factors that contributed to loss of life. Masskant [10] found that the consideration of the exact spatial distribution of population growth is essential for reliable estimation of future risk of flooding.

This paper mainly focuses on the risk analysis of earth-rock dams from the probability and life loss model of the dam failure [11,12,13]. In the aspect of dam failure probability, the traditional probability calculation only considers the randomness of the event occurrence and depends on the experience of the experts, but the factors that affect the dam failure are often complicated and fuzzy [14]. Therefore, an event tree analysis (ETA) method based on fuzzy set theory is proposed. Dam failure may cause heavy fatalities, property damage, and environmental deterioration. After calculating the dam failure probability, we also need to analyze the possible life loss caused by the dam failure and establish a life loss assessment model which is suitable for China, so as to serve the dam risk analysis and management.

The establishment of a dam failure probability and a dam failure life loss model is of great significance to reduce the failure risk, improve the ability to deal with sudden break time, reduce life loss, and improve the management level of dams.

## 2. Causes, Modes and Paths of Dam Failure

Obviously, analysis of dam failures is of critical importance for disasters prevention and mitigation. Hence, an insightful understanding of the characteristics of dam failures (e.g., failure causes, modes, and paths) is needed [15,16].

In different countries and regions, the characteristics and laws of dam failure patterns and dam failure possibilities are different. Therefore, dam failure history information in a certain region is of particular significance for the risk analysis of dams in this region. Through the statistical analysis of the history of dam breakage cases, summarizing the causes and modes of dam failure is highly necessary for reservoir dam risk assessment and emergency measures [17].

### 2.1. Statistical Analysis of Dam Failure Data

At present, many countries in the world have a large number of reservoir dams. Among them, many dams have caused heavy loss of life due to dam failure, as shown in Table 1 [18,19]. These dam failure events have shocked the world and should be studied in-depth.

Since the 1960s, there have been many serious dam failure events in China [20], as shown in Table 2. We should investigate the dam failure events that have caused heavy loss of life, clarify the situation of life loss, and summarize its rules. It can be used for significant reference to estimate the fatalities reasonably and to reduce life loss in the future.

**Figure 1:**
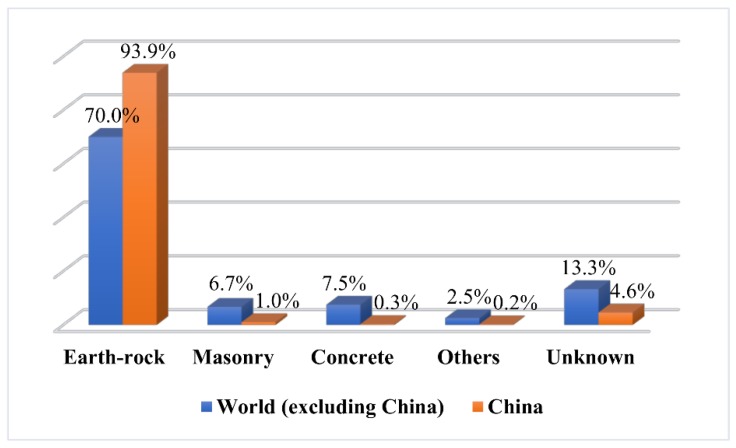
Statistics of dam types of the world (excluding China) and China.

According to the statistics in [21], the dam failure cases comprise earth dams, concrete dams, masonry dams, rockfill dams, and so on. Figure 1 compares the percentages of these types of dams in the world (excluding China) and in China. It clearly shows that the majority of cases are earth-rock dams, which account for 70.0% of dams in the world (excluding China) and 93.9% in China. Therefore, this paper chooses an earth-rock dam for failure analysis.

Earth-rock dam, one of the oldest dame types, generally refers to a dam that is constructed with local soil, stone, or mixture through throwing and rolling, etc. Such a dam has various risks in the operation, while dam failure is the most critical. Due to the complexity of the hydrogeographical environment, meteorology, hydrodynamic forces and structure of the dam, many factors can lead to dam failure. Therefore, it is very important to study the causes, modes, and paths in the earth-rock dam failure probability calculation.

### 2.2. Causes and Modes of Earth-Rock Dam Failure

**Figure 2:**
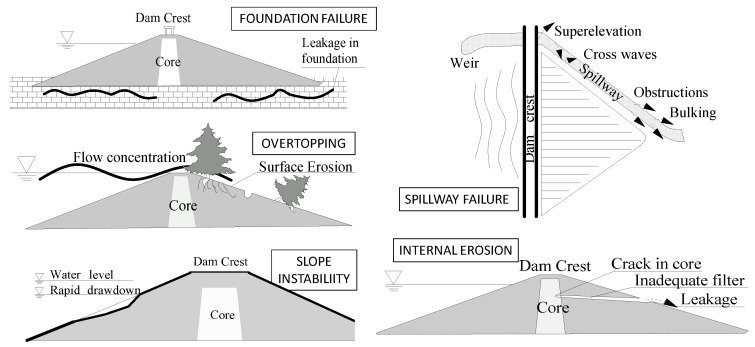
Several failure modes of earth-rock dams.

The earth-rock dam is the dam type with the largest number of calamities and highest dam failure rate. According to the failure mechanism, these can be divided into several types: lack of flood control capacity, insufficient structural stability, seepage damage, and several other conditions. The main failure modes are dam foundation failure, overtopping, slope instability, spillway failure, and internal erosion, as shown in Figure 2 [22,23,24,25].

## 3. Establishment of Analysis Models

### 3.1. Event Tree Analysis (ETA)

Event tree analysis (ETA) is a logic method, either qualitative and quantitative, that is used to identify possible outcomes. ETA is widely used as a ‘pre-accident’ analysis technique that examines the systems in place, which would prevent accident precursors from developing into accidents. It can also be used as a ‘post-accident’ analysis technique that identifies consequences of an accident sequence. The application of the ETA method in dam safety, where accident initiation is postulated, is used to illustrate how various subsequent events and scenarios evolve [26].

**Figure 3:**
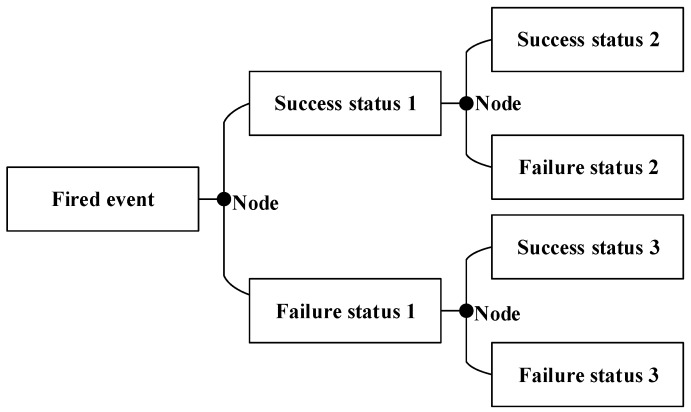
Event tree analysis of dam failure.

The risk analysis of dams with the ETA method is based on a condition or load condition. By using the tracing method, the various elements of the dam and how the failure happened under the load condition are logically analyzed. Thus, the estimation of overall dam failure probability is available. The ETA method is constructed as shown in Figure 3, which attempts to generate all the resultant events caused by some excitation events such as earthquake, flood, and internal defect: 

The ETA method provides a logical and graphical means to illustrate the sequence of events from an initiating event to the complete set of possible outcomes. The risk analysis of dam failure with the ETA method can help us to not only understand the overall process of the system change and identify possible accidents in advance, but also take measures to effectively avoid or reduce the incidence of accidents. According to the frequency of each risk factor occurrence, the probability of dam failure can be calculated.

However, the ETA method sometimes has less objective basis in estimating the probability of the event in every link of dam failure development. There is little historical data available for reference, most of which requires the experience of experts. The factors affecting dam failure are complicated and often fuzzy. In view of this, this paper also takes the fuzzy set theory into consideration to analyze the risk of earth-rock dam failure.

### 3.2. Fuzzy Set Theory

The concept of fuzzy set was proposed by Zadeh in 1965. The fuzzy set denotes the set of characteristic things with uncertain boundary [27]. For a fuzzy set *A* on the final field *X*, there is a $\mu_{A}(x)\in \left[0,1\right]$ corresponding to ∀x∈A. $\mu_{A}(x)$ is the membership degree of *x* to *A* and $\mu_{A}$ is the membership function of fuzzy set *A*.

#### 3.2.1. Concept of Fuzzy Numbers

Fuzzy numbers are used to deal with some fuzzy and inaccurate information, such as the “very likely” and “unlikely” language used by experts in the risk analysis of earth-rock dams which need to be quantified with fuzzy numbers combined with membership functions. In this paper, fuzzy numbers are divided into triangular fuzzy numbers and trapezoidal fuzzy numbers [28,29].

A triangular fuzzy number is expressed as A=(a,b,c), whose membership function is

(1) $$\mu_{A}(x)=\{\begin{array}{cc} 0; & x\leq a\\ \left(x-a)/(b-a\right) & a<x\leq b\\ \left(c-x)/(c-a\right) & b<x\leq c\\ 0 & x>c \end{array}$$

A trapezoidal fuzzy number is expressed as A=(a,b,c,d), whose membership function is

(2) $$\mu_{A}(x)=\{\begin{array}{cc} 0; & x\leq a\\ (x-a)/(b-a); & a<x\leq b\\ 1; & b<x\leq c\\ (d-x)/(d-c); & c<x\leq d\\ 0; & x>d \end{array}$$

#### 3.2.2. Operation of Fuzzy Numbers

For a given number ∀λ∈[0,1], the λ-cut of fuzzy set *A* and *B* can be expressed as

(3) $$\begin{array}{c} A^{\lambda }=\left\{x|x\in R,\mu_{A}\geq \lambda \right\}=[a_{1}^{\lambda },b_{1}^{\lambda }]\\ B^{\lambda }=\left\{x|x\in R,\mu_{B}\geq \lambda \right\}=[a_{2}^{\lambda },b_{2}^{\lambda }] \end{array}$$

Then the operation between fuzzy sets can be achieved by their λ-cut sets:

(4) $$\begin{array}{c} A(+)B=A^{\lambda }+B^{\lambda }=[a_{1}^{\lambda }+a_{2}^{\lambda },b_{1}^{\lambda }+b_{2}^{\lambda }]\\ A(-)B=A^{\lambda }-B^{\lambda }=[a_{1}^{\lambda }-a_{2}^{\lambda },b_{1}^{\lambda }-b_{2}^{\lambda }]\\ A(\times )B=A^{\lambda }\times B^{\lambda }=[a_{1}^{\lambda }\times a_{2}^{\lambda },b_{1}^{\lambda }\times b_{2}^{\lambda }]\\ A(÷)B=A^{\lambda }÷B^{\lambda }=[a_{1}^{\lambda }÷a_{2}^{\lambda },b_{1}^{\lambda }÷b_{2}^{\lambda }] \end{array}$$

#### 3.2.3. Non-Fuzzification of Fuzzy Language of Experts

**Figure 4:**
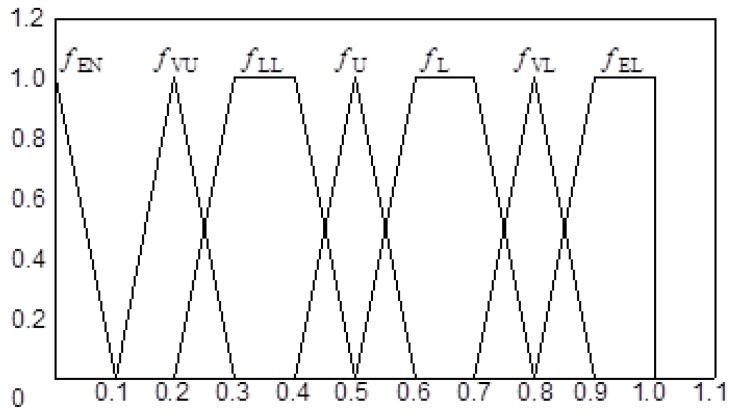
Membership function of fuzzy language.

For the ETA of dam failure, experts often use fuzzy language for qualitative evaluation. Usually, we need to transform the fuzzy language of experts into quantitative analysis and then evaluate the safety of reservoir dams comprehensively. For the probability of dam failure, the fuzzy language can be divided into seven types: ‘Extremely unlikely’, ‘Very unlikely’, ‘Less likely’, ‘Uncertain’, ‘Likely’, ‘Very Likely’, and ‘Extremely Likely’. The fuzzy numbers and corresponding λ-cut sets of them are expressed in Table 3 [30]:

The membership function is expressed in Figure 4: 

Meanwhile, in the process of organizing experts’ empowerment analysis, it is also necessary to analyze the credibility of different experts. In this paper, the credibility of the expert, also known as the weight of experts, is expressed as α (0<α<1). α=1 represents the expert being the most trusted and α=0 represents the expert being the least trusted. In this paper, the credibility of the expert is determined from four aspects: educational degree, professional title, professional direction, and length of service. The criteria for determining the credibility of an expert are shown in Table 4:

If $\beta_{j}$(*j* =1,2,3,4) is used to represent the evaluation scores of experts in four aspects: educational degree, professional title, professional direction and length of service, the credibility of each expert can be expressed as follows:

(5) $$\alpha_{i}=\sum_{j=1}^{4}\beta_{j}/40$$

#### 3.2.4. Integral Value Method of Non-Fuzzification for Fuzzy Number

After obtaining the corresponding λ-cut sets of fuzzy numbers, the integral value method proposed by Liou is used to calculate the fuzzy numbers [31].

(6) $$I^{\alpha }=\alpha I_{R}(A)+(1-\alpha )I_{L}(A)$$

where α is the index of optimism, α∈[0,1]. $I_{L}(A)$ and $I_{R}(A)$ are the inverse function of left and right integral values of *A* respectively.

(7) $$\begin{array}{c} I_{L}(A)=0.5\left[\sum_{\lambda =0.1}^{1}\lambda_{u}(A)\Delta \lambda +\sum_{\lambda =0}^{0.9}\lambda_{u}(A)\Delta \lambda \right]\\ I_{R}(A)=0.5\left[\sum_{\lambda =0.1}^{1}\lambda_{l}(A)\Delta \lambda +\sum_{\lambda =0}^{0.9}\lambda_{l}(A)\Delta \lambda \right] \end{array}$$

where $\lambda_{u}(A)$ and $\lambda_{l}(A)$ is upper bound and lower bound of λ-cut sets of *A*. The upper and lower bounds of the fuzzy number *A* are respectively corresponding to α=0 and α=1. The value of the fuzzy number is representative when α=0.5.

### 3.3. Application of Dam Failure Risk Analysis

The application of the ETA method based on fuzzy set theory in dam failure risk analysis of an earth-rock dam can be summed up in the following steps [32]:

(1) In view of the probable dam-breaking event, analyze the various accident paths and the accident links of the dam and establish the event tree structure chart.
(2) Calculate the probability of dam failure for all dam-breaking paths under each load condition.

Invite experts to carry out a qualitative assessment of the accident. By using the ETA method of fuzzy set theory, the expert qualitative language is converted into quantitative value. The probability of each failure link in the accident path is obtained. Finally, the probability value of the dam in a burst mode is obtained by multiplying the probabilities of each failure link. The conditional probability of each link in a burst mode is p(i,j,k), i=1,2,⋯,m; j=1,2,⋯,n; k=1,2,⋯,s. where *i* is the reservoir water level load, *j* is the failure mode and *k* represents for each aspect. Then, the probability of burst under the I type load and j type failure mode is

(8) $$P(i,j)=\prod_{k=1}^{s}p(i,j,k)$$

(3) Under the same load, the failure mode of the dam can be independent, at which time the deMorgan law can be used to calculate the burst probability under the same load.
  (9) $$P(A_{i1}+A_{i2}+\cdots +A_{im})=1-\prod_{j=1}^{m}\left(1-P_{ij}\right)$$
(4) Repeat the above steps for different loads on the dam to obtain all possible dam-breaking paths and their probabilities under all possible load conditions. Assuming that the dam failures under different loads are independent of each other, the probability of the dam collapse under all different loading conditions is added, that is, the total dam probability of the dam is obtained.
  (10) P=P(1)+P(2)+⋯+P(n)

### 3.4. Estimation Model of Dam Failure Life Loss

Evaluating the consequences of a dam failure is extremely important in the dam safety study. Dam failure can cause catastrophic losses such as life loss, property loss, environmental loss, and so on. The most important part is the loss of life. This paper focuses on estimating fatalities of a dam failure.

The estimation of dam failure life loss is affected by many factors [9]. Among these are cause and type of dam failure; number of people at risk; severity of dam break flood; timeliness of dam failure warnings; occurrence time of dam failure; ease of evacuation.

Graham summarized the seven basic steps to evaluate the dam failure life loss, which are still widely used at present. Ke-fa et al. [33,34] conducted an in-depth discussion and analysis on a large amount of data of eight dam failures that have occurred in the history of China. They summarized the basic law of the loss of life and proposed a life loss estimation method, which is suitable for Chinese conditions. In this paper, based on this method, combined with the actual situation of the studied reservoir, the potential loss of life caused by the dam failure will be evaluated.

#### 3.4.1. Estimation Model Parameters of Dam Failure Life Loss

Dam-break loss of life is a result of complex factors, usually divided into population at risk (*P_AR_*), severity degree of dam break flood (*S_D_*), occurrence time of dam failure, warning time (*W_T_*), and understanding of *P_AR_* to *S_D_*.

(1) Population at risk (*P_AR_*)

Population at risk refers to the number of people in the area covered by the dam-breaking flood. The larger the total population at risk is, and the closer it is to the dam site and main channel, the greater the resulting loss of life will be. *P_AR_* can be determined by survey statistics and population registration data:

(11) $$P_{AR}={\sum P_{AR}}_{i}$$

where *i* means a residential area and *P_ARi_* means population of the residential area.

When considering the *P_AR_*, other factors such as population composition, living environment, escape route, and emergency rescue capability should also be taken into consideration in order to obtain more appropriate results.

(2) Severity degree of dam failure flood (*S_D_*)

*S*_D_ refers to the damage degree of dam failure flood to the downstream residents and buildings, which is related to dam type, storage capacity, and discharging flow. *S_D_* is usually represented by the *D* × *V* value of the water depth and the velocity of a section:

(12) $$S_{D}=\{\begin{array}{ccc} \mathrm{Low} & \mathrm{severity}, & D\times V<1.0m^{2}/s\\ \mathrm{Medium} & \mathrm{severity}, & 1.0m^{2}/s\leq D\times V\leq 4.0m^{2}/s\\ \mathrm{High} & \mathrm{severity}, & D\times V>4.0m^{2}/s \end{array}$$

(3) Warning time (*W_T_*)

Warning time refers to the time from the moment of the dam failure warning to the time when the downstream masses retreated after receiving the instruction. It has an important influence on the amount of loss of life. *W_T_* can generally be divided into three categories:

(13) $$\{\begin{array}{ccc} \mathrm{Little} & \mathrm{warning}, & W_{T}<0.25h\\ \mathrm{Partly} & \mathrm{warning}, & 0.25h\leq W_{T}\leq 1.0h\\ \mathrm{Full} & \mathrm{warning} & W_{T}>1.0h \end{array}$$

(4) Occurrence time (*O_T_*)

Occurrence time of dam failure has a significant impact on the *P_AR_* and *W_T_*. According to the weather, occurrence time can be divided into sunny and rainy days; according to the time of day, it can be divided into daytime and night; according to the season, it can be divided into winter and summer.

*O_T_* is very important in the evaluation of life loss. If the dam break occurs on sunny days the traffic will be better; during the daytime, it is more easily found by the staff; if the dam break occurs in summer, it is good for the evacuation of *P_AR_*.

(5) Understanding of *P_AR_* to *S_D_* (*U_D_*)

Understanding of *S_D_* for *P_AR_* will affect the success rate of rescue methods, which is an important aspect in estimation of in dam failure life loss. *U_D_* can be divided into two types: (1) *U_D_* is fuzzy: the population at risk cannot understand the severity degree of the dam break flood when they get the warning and they do not know the necessity, measure, and path of escape. (2) *U_D_* is explicit: the population at risk can understand the severity degree of the dam break flood clearly and can take the necessary measures of escape clearly.

#### 3.4.2. Calculation of Estimation Model of Dam Failure Life Loss

Based on the Graham method and combined with the situation of dams in China, this paper adopts the method of estimating the loss of life of dam failure in China proposed by Lei [33].

This calculation model mainly considers three parts: the number of population at risk (*P_AR_*), the risk mortality rate suitable for China *f*, and the corresponding correction coefficient ω. The formula is as follows:

(14) $$L_{OL}=\omega \times P_{AR}\times f$$

where the value of *f* is determined according to Table 5.

Remarks: when it is sunny daytime, the upper limit is recommended and when it is rainy night, the lower limit is recommended.

## 4. Engineering Examples

### 4.1. Project Introduction

#### 4.1.1. Project Overview

Located in the upper reaches of the Zhangjiang River, Ganzhou city, Jiangxi province, a hydropower project is large (2) type of water conservancy project dominated by flood control [35]. Reservoir total capacity is 1.19 × 10^8^ m^3^ at the normal pool level of 220.00 m. The checked flood level is 223.70 m and dead water level is 209.00 m.

In March 2004, the dam was identified as a third-type dam, which would be reinforced in 2008. This paper analyzed the probability of dam failure and the life loss model based on the data before the reinforcement work, aiming to analyze the risk of dam failure before reinforcement and to improve the safety management level. This analysis will provide a significant reference for other dam analyses.

**Figure 5:**
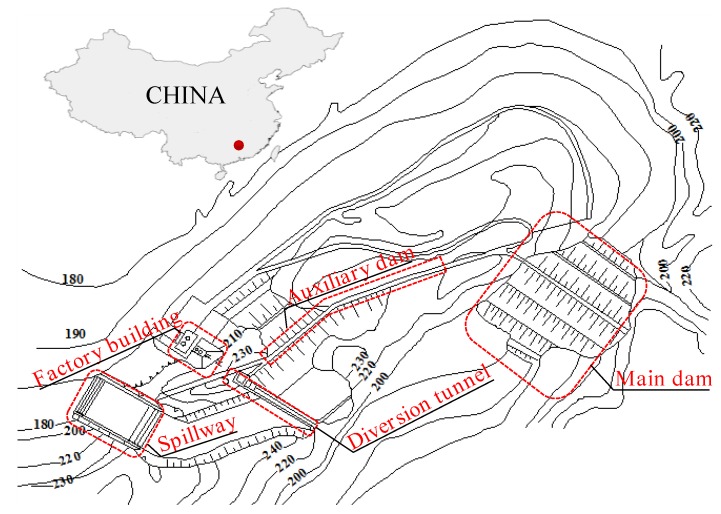
Layout plan of the reservoir project.

**Figure 6:**
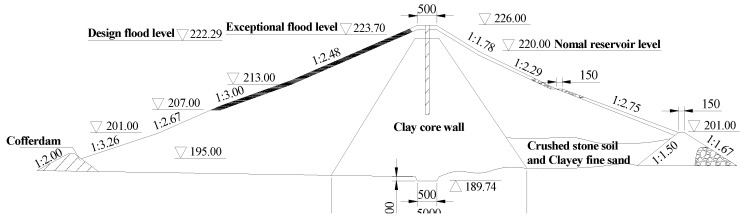
Typical cross-section structure dimensions of the dam.

The layout plan of the reservoir project is shown in Figure 5:

The dam is a roller thick clay core earth dam with a crest elevation of 226.0 m, a maximum dam height of 36.0 m, a crest width of 5.0 m, and a crest length of 177.0 m. The dam typical cross-section structure dimensions are shown in Figure 6.

#### 4.1.2. Main Problems of the Dam

(1) Main dam: Severe leakage in the dam body, prominent by-pass seepage, severely weathered slope protection rock with the danger of landslide.
(2) Auxiliary dam: Existence of permeable layer because of the deficient foundation clearance, the probability of infiltration and damage of left bank, weak anti-seepage function of inclined wall.
(3) Spillway: Serious erosion, cracks and tendons in the spillway pier and concrete shaft of the spillway, seriously deterioration of gates and electrical facilities.

### 4.2. Analysis of Dam Failure Probability

#### 4.2.1. Dam Failure Modes and Paths

According to former data, the failure of earth-rock dams in China is mainly based on three conditions: (1) Failure of the dam structures caused by the water load in non-flood season, such as seepage failure; (2) Serious floods in flood season which cause dam collapse, such as overtopping, seepage damage, slope landslides, and so on; (3) The dam collapse caused by an earthquake, such as seepage damage, structural failure, and so on. As for the dam area reference, the basic earthquake intensity is less than 6 degrees in this area. This paper does not consider the dam failure caused by the earthquake load according to the relevant norms.

Aiming at the dam failure caused by the water load in flood season and non-flood season, this paper screens and analyzes all the dam failure modes and get the main failure modes and damage paths as follows [36,37,38]:

(1) Non-flood season load
  Leakage of main dam foundation–Piping–Manual intervention–Invalidation of intervention–dam failure;
  Leakage of auxiliary dam foundation–Piping–Manual intervention–Invalidation of intervention–dam failure;
  By-pass seepage of auxiliary dam shoulder–Piping–Manual intervention–Invalidation of intervention–dam failure;
(2) Flood season load
  Flood–Leakage of main dam foundation–Piping–Manual intervention–Invalidation of intervention–dam failure;
  Flood–Leakage of auxiliary dam foundation–Piping–Manual intervention–Invalidation of intervention–dam failure;
  Flood–By-pass seepage of auxiliary dam shoulder–Piping–Manual intervention–Invalidation of intervention–dam failure;
  Flood–By-pass seepage of auxiliary dam sloping core–Piping–Manual intervention–Invalidation of intervention–dam failure;
  Flood–Failure of spillway structure–Breach expanded–Manual intervention–Invalidation of intervention–dam failure;

#### 4.2.2. Calculation of Dam Failure Probability

The reservoir is designed according to the 500-year flood (*P* = 0.2%) and checked according to the 5000-year flood (*P* = 0.02%). When we select the characteristic load value of the dam, we select the normal water level 220.00 m as the non-flood load value with the frequency of 1.0 and the check flood level 223.70 m as the flood load with the frequency of 0.02%.

The key to estimating the risk rate of dam failure by using the ETA method of fuzzy set theory is to calculate the probability of each link accident.

According to the dam failure mode and the dam break path, the event tree of the dam failure is constructed. This paper takes the case of the piping of the auxiliary dam shoulder under the condition of 223.70 m water level in flood season as an example.

**Figure 7:**
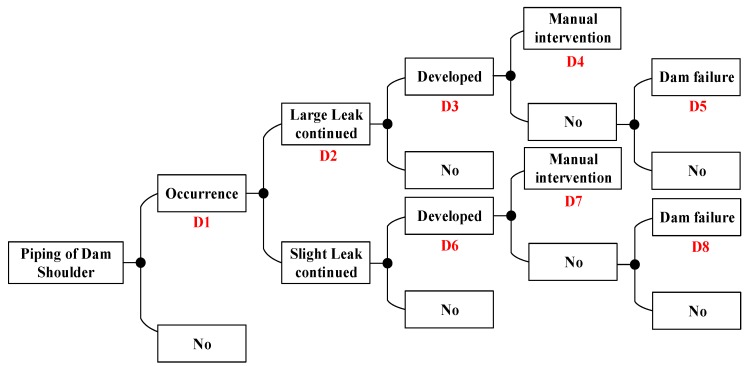
Piping of auxiliary dam shoulder event tree in the flood season.

Combined with the judgment of five experts (E1, E2, E3, E4, E5), the probabilities of each part of dam failure under this condition are calculated in Figure 7.

The five experts respectively evaluated D1, D2, D3, D4, D5, D6, D7, and D8 of each link of the piping event tree of the auxiliary dam shoulder [39]. The fuzzy probabilities are shown in Table 6.

Considering that different experts have different understandings of the actual operation status of the dam, there are differences among experts in terms of their level of knowledge, professional standards, personal experience ability, and other factors. In order to reduce the influence of expert subjectivity on the calculation results, this paper uses the weight coefficient of five experts to revise the evaluation and get the final evaluation results [40].

(15) $$P_{i}=\frac{\sum_{j=1}^{m}\omega_{ij}E_{ij}}{\sum_{j=1}^{m}\omega_{ij}}$$

where *P_i_* represents result of the expert’s comprehensive evaluation of event *i*, $\omega_{ij}$ represents the weighting coefficient of the event *i* of the *j*-th expert’s evaluation, and *E_ij_* represents the evaluation result of the *i*-th event by the *j*-th expert.

The weight coefficient of experts can be calculated by 1~9 scale judgment matrix, and the judgment matrix must meet the consistency requirement, otherwise it should be rebuilt. The judgment matrix of experts are shown in Table 7.

The estimated values of the fuzzy numbers in each link of the auxiliary dam shoulder are respectively:

$$P_{1}^{\lambda }=0.283E_{11}+0.168E_{12}+0.123E_{13}+0.073E_{14}+0.353E_{15}=\left(0.1\lambda +0.424,-0.1\lambda +0.648\right)$$

$$P_{2}^{\lambda }=(0.1\lambda +0.436,-0.1\lambda +0.671)$$

$$P_{3}^{\lambda }=(0.1\lambda +0.337,-0.1\lambda +0.579)$$

$$P_{4}^{\lambda }=(0.1\lambda +0.441,-0.1\lambda +0.681)$$

$$P_{5}^{\lambda }=(0.1\lambda +0.489,-0.1\lambda +0.742)$$

$$P_{6}^{\lambda }=(0.1\lambda +0.329,-0.1\lambda +0.564)$$

$$P_{7}^{\lambda }=(0.1\lambda +0.257,-0.1\lambda +0.528)$$

$$P_{8}^{\lambda }=(0.1\lambda +0.164,-0.1\lambda +0.427)$$

The calculation result is substituted by the Formulas (6) and (7) to calculate the fuzzy numbers. By adopting the integral value method proposed by Liou to calculate the fuzzy numbers, the fuzzy probability of each link of the auxiliary dam shoulder is obtained. Where α=0 and α=1 respectively correspond to the upper and lower bounds of the fuzzy numbers of the failure probability *P_L_* and *P_U_*. When α=0.5, the calculated value obtained is the probability value *P* of the accident link. The following Table 8 is available:

The probability of the auxiliary dam shoulder failure is:

$$P=P_{L\arg e}+P_{Slight}=P_{D1}\cdot P_{D2}\cdot P_{D3}\cdot P_{D4}\cdot P_{D5}+P_{D1}\cdot (1-P_{D2})\cdot P_{D6}\cdot P_{D7}\cdot P_{D8}=0.059$$

Considering the occurrence frequency of a flood season load of 223.70 m water level is 0.02%, the risk rate of the earth-rock dam with the water level of 223.70 m in the flood season and the dam failure occurring is:

$$f\times P=0.059\times 0.0002=1.18\times 10^{-5}$$

#### 4.2.3. Calculation of Dam Failure under Different Load Conditions

According to the method above, the dam failure probabilities occurring under other conditions can be obtained, which are shown in Figure 8, Figure 9, Figure 10, Figure 11, Figure 12, Figure 13, Figure 14 and Figure 15.

According to the above ETA method based on fuzzy set theory, we can summarize the dam failure probabilities under non-flood season and flood season load in Table 9:

The probability of the dam failure is 1.16 × 10^−4^, which is higher than the maximum probability of collapse estimated by the Bureau of Reclamation 10^−4^; thus, measures should be taken to reduce the failure probability. Table 9 shows that the main dam failure mode is the main dam foundation piping and the auxiliary dam foundation piping. The analysis results are consistent with the actual conditions of the dam. In view of the analysis results, we should focus on strengthening the reinforcement of main dam foundation and auxiliary dam foundation. During the flood season, strengthening the monitoring of flood data and paying attention to the reinforcement of the spillway are necessary.

### 4.3. Estimation Model of Dam Failure Life Loss

Dayu County, a total area of 1367.63 km^2^, administers 11 townships. In 2006, the county’s resident population was 291,969, with an average population density of 214 persons/km^2^. The population and the buildings are dense.

#### 4.3.1. Determination of Influencing Factors

(1) Population at risk (*P_AR_*)

First, identify the population at risk (*P_AR_*). According to the flooding range of dam break floods, the following Table 10 is obtained according to the government statistics on the population distribution of the submerged area. Among them, the number of *P_AR_* in the daytime and night are different; the impact factors are 0.5 and 0.8 respectively.

(2) Severity degree of dam break flood (*S_D_*)

Calculate the average *D* × *V* value of the submerging range’s administrative area, as shown in the following Table 11:

(3) Warning time (*W_T_*)

The calculated area is relatively small, with only Fujiang township and Nan’an Town. Modern communications are more developed. Therefore, it is assumed that the flood warning time (*W_T_*) is the same, divided into: 0~0.25 h, 0.25 ~1.0 h, and beyond 1.0 h.

(4) Occurrence time (*O_T_*)

This simulated dam break occurs only in the daytime and night conditions, and other conditions such as weather and season are temporarily not taken into consideration.

(5) Understanding of *P_AR_* to *S_D_* (*U_D_*)

The understanding of *P_AR_* to *S_D_* (*U_D_*) is shown is Table 12.

#### 4.3.2. Estimation of Dam Failure Life Loss

The formula of estimation of dam failure loss of life (*L_OL_*) is as follows:

(16) $$L_{OL}=\omega \times P_{AR}\times f$$

where *f* is according to the Figure 5. For the dam considered in this paper, the corresponding correction coefficient ω is as follows according to the relevant information, which is shown in Table 13.

Then, we can make the estimation according the formula above. The results of the estimation of dam failure loss life are shown below.

In general, 

Combined with the calculation results from 3.2, the dam has a 1.16 × 10^−4^ probability of dam failure. The possible loss of life in this case is estimated as shown in Table 18.

As can be seen from the Table 14, Table 15, Table 16,Table 17 and Table 18, the life loss (*L_OL_*)of dam failure is most affected by the warning time (*W_T_*), and the occurrence time (daytime or night) also has great influence. Therefore, the dam workers should strengthen safety monitoring (especially in flood season) and improve the warning time. It is highly necessary to ensure *W_T_* is longer than one hour, which can significantly reduce the loss of life of dam failure. According to the social risk standard *F-N* reference map of China reservoir dams, the calculated risk of life loss is higher than 1.1 × 10^−3^, which is an intolerable risk. So the dam reinforcement project should also be carried out, which is consistent with the 3.2 research conclusion.

In the event that dam engineering facilities cannot be changed and dam-breaking floods cannot be perfectly predicted, we need to strengthen the management of non-engineering measures in order to reduce the dam failure life loss. Some suggested actions below can be taken into consideration:

(1) Evacuate residents in times of flood season and minimize the risk to the population;
(2) Strengthen the monitoring work (especially in the flood season), improve the capability of early warning of dam breakage, and increase the warning time, which must be more than one hour;
(3) Strengthen the liaison among downstream residents to ensure smooth communication;
(4) Improve the level of contingency plans, ensure the availability of traffic in the submerged area and enhance the rescue capability after dam failure.

## 5. Conclusions

This paper conducted the risk analysis of an earth-rock dam failure, which involves several complicated factors. Based on the analyzed results, the following can be concluded.

(1) After considering various factors affecting the dam safety, a new model with the ETA method based on fuzzy set theory is proposed for the dam failure analysis, and good results were achieved. By using the ETA method, the probability of each link of dam failure mode can be clearly evaluated. Combined with fuzzy set theory, experienced experts are invited to carry out the evaluation and analysis. The main failure modes of the dam in non-flood season are the main dam and auxiliary dam piping, and in flood season the spillway failure needs to be considered. The project reinforcement should be carried out in a targeted manner.
(2) After considering other scholars’ estimation model of life loss, population at risk (*P_AR_*), severity degree of dam break flood (*S_D_*), warning time (*W_T_*), occurrence time (*O_T_*) and understanding of *P_AR_* to *S_D_* (*U_D_*) are taken into consideration as well as the corresponding correction coefficient ω. The new estimation model is applied to a specific project and the expected dam failure life loss exceeds the standard requirements. Some non-engineering measures are proposed with a view of reducing the dam failure life loss.

In summary, the dam risk is already at a high stage. It is highly necessary to reinforce the project in addition to strengthening the flood season monitoring and raising the warning time.

The dam failure risk analysis model proposed in this paper has been successfully applied to a specific water conservancy project and achieved good results. For other dams, the analysis model can also be applied to improve the operation of dam management.

## Figures and Tables

**Figure 8:**
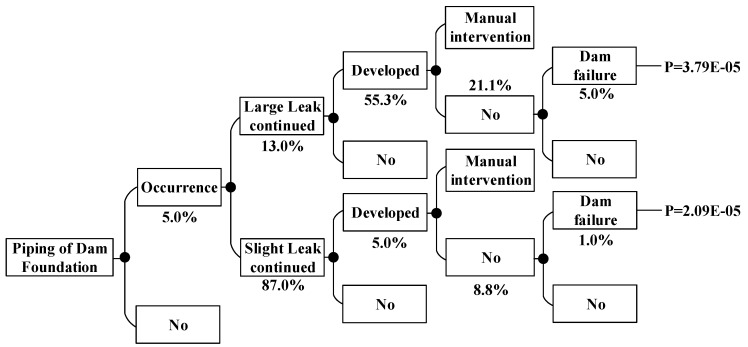
Piping of main dam foundation event tree in the non-flood season.

**Figure 9:**
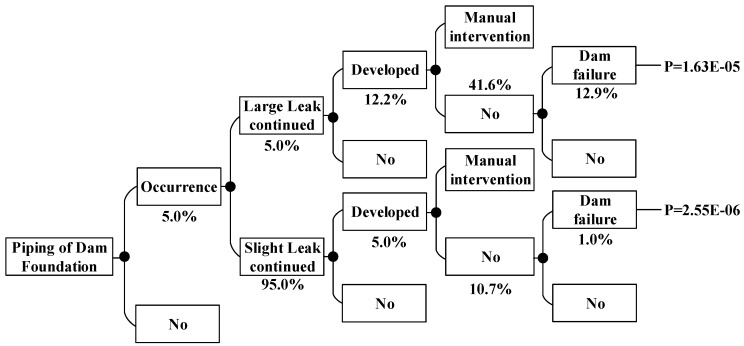
Piping of auxiliary dam foundation event tree in the non-flood season.

**Figure 10:**
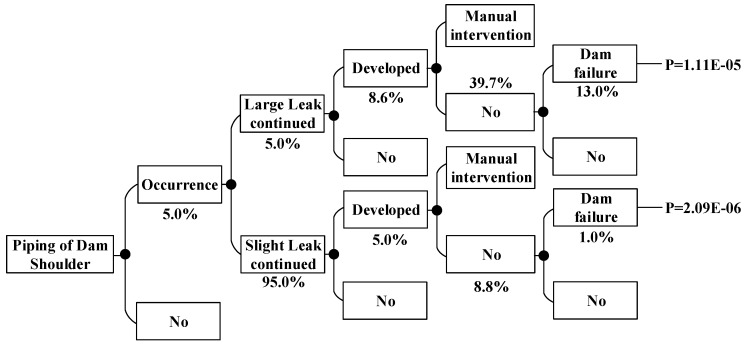
Piping of auxiliary dam shoulder event tree in the non-flood season.

**Figure 11:**
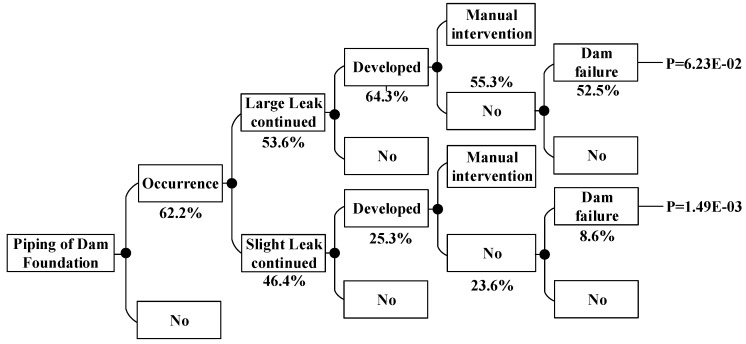
Piping of main dam foundation event tree in the flood season.

**Figure 12:**
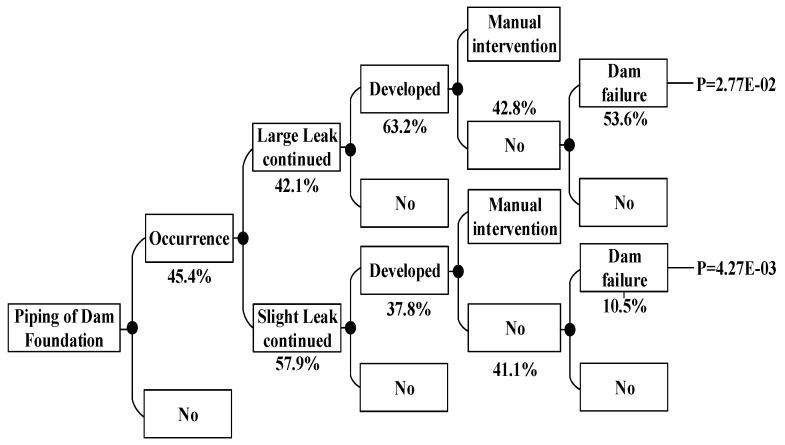
Piping of auxiliary dam foundation event tree in the flood season.

**Figure 13:**
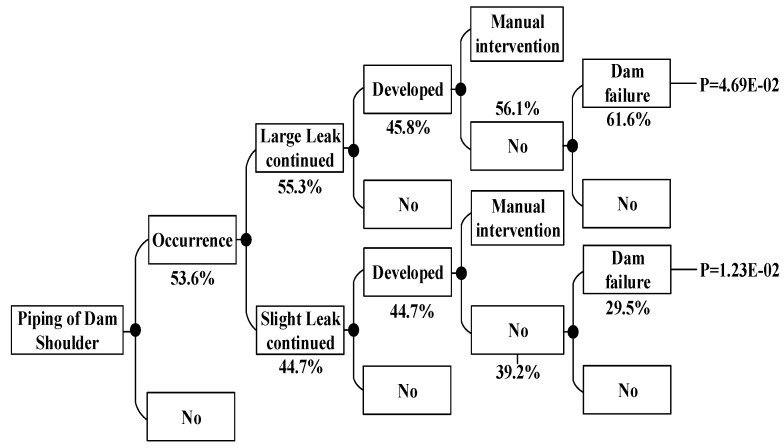
Piping of auxiliary dam shoulder event tree in the flood season.

**Figure 14:**
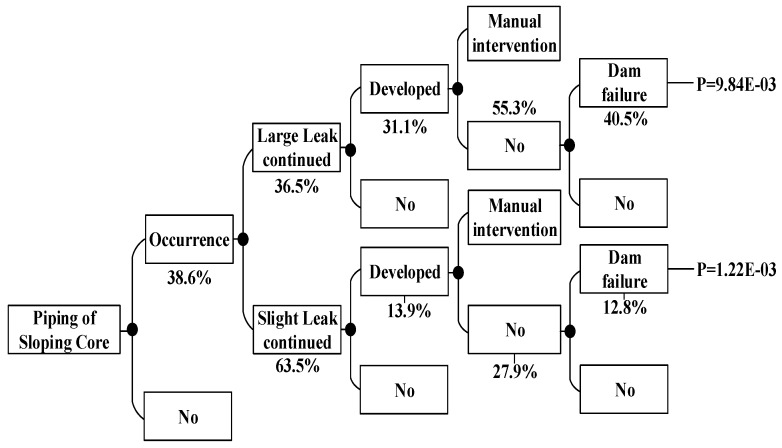
Piping of sloping core event tree in the flood season.

**Figure 15:**
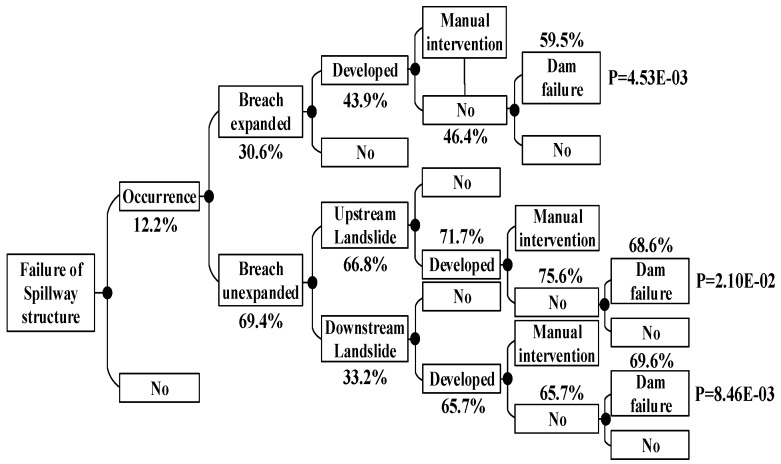
Failure of spillway structure core event tree in the flood season.

**Table 1:** Several famous large dam failure events and deaths in the world.

Dam Name	Country	Year of Accident	Dam Type	Reservoir Volume (×10^6^ m^3^)	Deaths
Mohne Dam	German	1943	Gravity dam	134.0	1200
Malpasset Dam	France	1959	Arch dam	15.0	421
Vaiont Dam	Italy	1963	Arch dam	169.0	2000
Buffalo Creek Dam	USA	1972	Tailings dam	49.8	125
Machhu II Dam	India	1979	Earth dam	101.0	3000
Shakidor Dam	Pakistan	2005	Earth-rock dam	-	135
Situ Gintung Dam	Indonesia	2009	Earth-rock dam	2.0	100

**Table 2:** Several large dam failure events and deaths in China.

Dam Name	Location	Date	Dam Type	Reservoir Volume (10^6^ m^3^)	Deaths
Longtun Dam	Suizhong, Liaoning province	1959.7.22	Clay sloping core dam	30.0	707
Liujiatai Dam	Yixian, Hebei province	1963.8.8	Clay core wall dam	40.5	943
Hengjiang Dam	Jiexi, Guangdong province	1970.9.15	Homogeneous earth dam	78.8	941
Lijiaju Dam	Zhuanglang, Gansu province	1973.4.29	Homogeneous earth dam	1.1	580
Banqiao Dam	Luoyang, Henan province	1975.8.8	Clay core wall dam	492.0	22,564
Shimantan Dam	Wugang, Henan province	1975.8.8	Homogeneous earth dam	91.8	
Gouhou Dam	Gonghe, Qinghai province	1993.8.27	Concrete-faced rock-fill dam	3.0	400

**Table 3:** Fuzzy numbers and corresponding λ-cut sets of fuzzy language.

Fuzzy Language	Fuzzy Number	λ-Cut Set
Extremely unlikely	f=(0,0,0.1)	$f^{\lambda }=(0,-0.1\lambda +0.1)$
Very unlikely	f=(0.1,0.2,0.3)	$f^{\lambda }=(0.1\lambda +0.1,-0.1\lambda +0.3)$
Less likely	f=(0.2,0.3,0.4,0.5)	$f^{\lambda }=(0.1\lambda +0.2,-0.1\lambda +0.5)$
Uncertain	f=(0.4,0.5,0.6)	$f^{\lambda }=(0.1\lambda +0.4,-0.1\lambda +0.6)$
Likely	f=(0.5,0.6,0.7,0.8)	$f^{\lambda }=(0.1\lambda +0.5,-0.1\lambda +0.8)$
Very likely	f=(0.7,0.8,0.9)	$f^{\lambda }=(0.1\lambda +0.7,-0.1\lambda +0.9)$
Extremely Likely	f=(0.8,0.9,1.0)	$f^{\lambda }=(0.1\lambda +0.8,1)$

**Table 4:** Criteria for determining the credibility of an expert.

**Aspects**	**Educational Degree**			**Professional Title**		
	Doctor	Master	Bachelor	Senior	Medium-grade	Junior
Scoring range	[8,10]	[7,9]	[6,8]	[8,10]	[7,10]	[5,7]
**Aspects**	**Professional direction**			**Length of service**		
	Hydraulic structure engineering	Hydropower engineering	Civil Engineering	>20a	10a~20a	<10a
Scoring range	[7,10]	[5,10]	[5,8]	[8,10]	[5,7]	[4,6]

**Table 5:** Risk mortality rate suitable for China *f.*

*S_D_*	*W_T_*(h)	*U_D_*	*f*	
			Recommended Average	Recommended Range
High	<0.25	Fuzzy	0.7500	0.3000~1.0000
		Explicit	0.2500	0.1000~0.5000
	0.25~1.0	Fuzzy	0.2000	0.0500~0.4000
		Explicit	0.0100	0.0050~0.0200
	>1.0	Fuzzy	0.1800	0.0100~0.3000
		Explicit	0.0005	0.0000~0.0010
Medium	<0.25	Fuzzy	0.5000	0.1000~0.8000
		Explicit	0.0750	0.0200~0.1200
	0.25~1.0	Fuzzy	0.1300	0.0150~0.2700
		Explicit	0.0008	0.0005~0.0020
	>1.0	Fuzzy	0.0500	0.0100~0.1000
		Explicit	0.0004	0.0002~0.0010
Low	<0.25	Fuzzy	0.0300	0.0100~0.0500
		Explicit	0.0100	0.0000~0.0200
	0.25~1.0	Fuzzy	0.0070	0.0000~0.0150
		Explicit	0.0006	0.0000~0.0010
	>1.0	Fuzzy	0.0003	0.0000~0.0006
		Explicit	0.0002	0.0000~0.0004

**Table 6:** Experts’ judgments of piping of auxiliary dam shoulder in the flood season.

	E1	E2	E3	E4	E5
D1	Uncertain	Likely	Uncertain	Likely	Uncertain
D2	Likely	Uncertain	Uncertain	Likely	Uncertain
D3	Uncertain	Uncertain	Uncertain	Likely	Less likely
D4	Likely	Uncertain	Likely	Uncertain	Uncertain
D5	Likely	Likely	Very likely	Likely	Uncertain
D6	Less likely	Uncertain	Uncertain	Less likely	Uncertain
D7	Uncertain	Less likely	Less likely	Less likely	Less likely
D8	Less likely	Very unlikely	Very unlikely	Very unlikely	Very unlikely

**Table 7:** Judgment matrix of experts.

Experts	E1	E2	E3	E4	E5	Weight Coefficient $\omega_{ij}$
E1	1	3	4	2	1/2	0.283
E2	1/3	1	2	4	1/3	0.168
E3	1/4	1/2	1	3	1/2	0.123
E4	1/2	1/4	1/3	1	1/3	0.073
E5	2	3	2	3	1	0.353

**Table 8:** Probabilities of each accident of the auxiliary dam shoulder.

Event	D1	D2	D3	D4	D5	D6	D7	D8
*P*	0.536	0.553	0.458	0.561	0.616	0.447	0.392	0.295
*P_L_*	0.474	0.486	0.387	0.491	0.539	0.379	0.307	0.214
*P_U_*	0.598	0.621	0.529	0.631	0.692	0.514	0.478	0.377

**Table 9:** Dam failure probabilities summary.

Load	Failure Mode and Parts	Frequency *f*	Failure Probability *P*	*f* × *P*	Percentage
non-flood season (220.00 m)	Piping of main dam foundation	100%	4.00 × 10^−5^	4.00 × 10^−5^	34.54%
	Piping of auxiliary dam foundation	100%	1.88 × 10^−5^	1.88 × 10^−5^	16.23%
	Piping of auxiliary dam shoulder	100%	1.32 × 10^−5^	1.32 × 10^−5^	11.40%
flood season (233.70 m)	Piping of main dam foundation	0.02%	6.38 × 10^−2^	1.28 × 10^−5^	11.05%
	Piping of auxiliary dam foundation	0.02%	5.93 × 10^−2^	1.18 × 10^−5^	10.28%
	Piping of auxiliary dam shoulder	0.02%	5.03 × 10^−2^	1.01 × 10^−5^	8.72%
	Piping of sloping core	0.02%	1.11 × 10^−2^	2.21 × 10^−6^	1.91%
	Failure of spillway structure	0.02%	3.40 × 10^−2^	6.80 × 10^−6^	5.87%
Total			1.16 × 10^−4^		

**Table 10:** *P*_AR_ of the submerged area.

Administrative Region	Households	Population	*P_AR_* (Daytime)	*P_AR_* (Night)
Fujiang township	1261	4727	2363	3782
Nan’an town	18,727	65,735	32,868	52,588
Total	19,988	70,462	35,232	56,370

**Table 11:** Average *D* × *V* value and corresponding *S_D_*.

Administrative Region	*D* × *V* (m^2^/s)	*S_D_*
Fujiang township	14.48	High
Nan’an town	11.26	High

**Table 12:** Understanding of *P_AR_* to *S_D_* (*U_D_*).

Warning Time	0~0.25 h	0.25~1.0 h	>1.0 h
Daytime	Fuzzy	Explicit	Explicit
Night	Fuzzy	Fuzzy	Explicit

**Table 13:** Corresponding correction coefficient ω .

Warning Time	0~0.25 h	0.25~1.0 h	>1.0 h
Daytime	0.80	0.60	0.40
Night	0.80	0.65	0.50

**Table 14:** Estimated dam failure loss of life when *W_T_* = 0~0.25 h.

Administrative Region		Fujiang Township	Nan’an Town
*DV* (m^2^/s)		14.48	11.26
*S_D_*		High	High
*W_T_*		0~0.25 h	0~0.25 h
Daytime	*P_AR_*	2363	32,868
	*U_D_*	Fuzzy	Fuzzy
	*F*	0.30	0.30
	ω	0.80	0.80
	*L_OL_*	567	7888
Night	*P_AR_*	3782	52,588
	*U_D_*	Fuzzy	Fuzzy
	*F*	0.70	0.70
	ω	0.80	0.80
	*L_OL_*	2118	29,449

**Table 15:** Estimated dam failure loss of life when *W_T_* = 0.25~1.0 h.

Administrative Region		Fujiang Township	Nan’an Town
*DV*(m^2^/s)		14.48	11.26
*S_D_*		High	High
*W_T_*		0.25~1.0 h	0.25~1.0 h
Daytime	*P_AR_*	2363	32,868
	*U_D_*	Explicit	Explicit
	*f*	0.01	0.01
	ω	0.6	0.6
	*L_OL_*	14	197
Night	*P_AR_*	3782	52,588
	*U_D_*	Fuzzy	Fuzzy
	*f*	0.2	0.2
	ω	0.65	0.65
	*L_OL_*	492	6836

**Table 16:** Estimated dam failure loss of life when *W_T_* > 1.0 h.

Administrative Region		Fujiang Township	Nan’an Town
*DV*(m^2^/s)		14.48	11.26
*S_D_*		High	High
*W_T_*		>1.0 h	>1.0 h
Daytime	*P_AR_*	2363	32,868
	*U_D_*	Explicit	Explicit
	*f*	0.001	0.001
	ω	0.4	0.4
	*L_OL_*	1	13
Night	*P_AR_*	3782	52,588
	*U_D_*	Explicit	Explicit
	*f*	0.001	0.001
	ω	0.5	0.5
	*L_OL_*	2	26

**Table 17:** Estimated dam failure loss of life.

ω		0~0.25 h	0.25~1.0 h	>1.0 h
*L_OL_*	Daytime	8455	211	14
	Night	31,567	7328	28

**Table 18:** Possible loss of life of the dam failure estimated.

ω		0~0.25 h	0.25~1.0 h	>1.0 h
*L_OL_*	Daytime	9.79 × 10^−1^	2.44 × 10^−2^	1.62 × 10^−3^
	Night	3.66	8.49 × 10^−1^	3.24 × 10^−3^
